# Natural products and long non-coding RNAs in prostate cancer: insights into etiology and treatment resistance

**DOI:** 10.1007/s00210-024-03736-x

**Published:** 2025-01-18

**Authors:** Hanan Elimam, Mohamed Bakr Zaki, Mai A. Abd-Elmawla, Hebatallah A. Darwish, Abdulrahman Hatawsh, Nora M. Aborehab, Sherif S. Abdel Mageed, Rewan Moussa, Osama A. Mohammed, Mustafa Ahmed Abdel-Reheim, Ahmed S. Doghish

**Affiliations:** 1 Department of Biochemistry, Faculty of Pharmacy, University of Sadat City, Sadat City, 32897 Egypt; 2 Department of Biochemistry, Faculty of Pharmacy, Cairo University, Cairo, Egypt; 3 Pharmacology, Toxicology and Biochemistry Department, Faculty of Pharmacy, Future University in Egypt, Cairo, Egypt; 4 Biotechnology School, Nile University, 26Th of July Corridor, Sheikh Zayed City, 12588 Giza Egypt; 5 Department of Biochemistry, Faculty of Pharmacy, Ahram Canadian University, Giza, Egypt; 6 Pharmacology and Toxicology Department, Faculty of Pharmacy, Badr University in Cairo (BUC), Badr City, 11829 Cairo Egypt; 7 School Faculty of Medicine, Helwan University, Cairo, 11795 Egypt; 8 Department of Pharmacology, College of Medicine, University of Bisha, 61922 Bisha, Saudi Arabia; 9 Department of Pharmacology, College of Pharmacy, Shaqra University, 11961 Shaqra, Saudi Arabia; 10 Department of Biochemistry, Faculty of Pharmacy, Badr University in Cairo (BUC), Badr City, , 11829 Cairo Egypt; 11 Biochemistry and Molecular Biology Department, Faculty of Pharmacy (Boys), Al-Azhar University, Nasr City, 11231 Cairo Egypt

**Keywords:** Prostate cancer, LncRNA, Chemotherapy, Diagnosis, Natural product

## Abstract

Globally, the incidence and death rates associated with cancer persist in rising, despite considerable advancements in cancer therapy. Although some malignancies are manageable by a mix of chemotherapy, surgery, radiation, and targeted therapy, most malignant tumors either exhibit poor responsiveness to early identification or endure post-treatment survival. The prognosis for prostate cancer (PCa) is unfavorable since it is a perilous and lethal malignancy. The capacity of phytochemical and nutraceutical chemicals to repress oncogenic lncRNAs and activate tumor suppressor lncRNAs has garnered significant attention as a possible strategy to diminish the development, proliferation, metastasis, and invasion of cancer cells. A potential technique to treat cancer and enhance the sensitivity of cancer cells to existing conventional therapies is the use of phytochemicals with anticancer characteristics. Functional studies indicate that lncRNAs modulate drug resistance, stemness, invasion, metastasis, angiogenesis, and proliferation via interactions with tumor suppressors and oncoproteins. Among them, numerous lncRNAs, such as HOTAIR, PlncRNA1, GAS5, MEG3, LincRNA-21, and POTEF-AS1, support the development of PCa through many molecular mechanisms, including modulation of tumor suppressors and regulation of various signal pathways like PI3K/Akt, Bax/Caspase 3, P53, MAPK cascade, and TGF-β1. Other lncRNAs, in particular, MALAT-1, CCAT2, DANCR, LncRNA-ATB, PlncRNA1, LincRNA-21, POTEF-AS1, ZEB1-AS1, SChLAP1, and H19, are key players in regulating the aforementioned processes. Natural substances have shown promising anticancer benefits against PCa by altering essential signaling pathways. The overexpression of some lncRNAs is associated with advanced TNM stage, metastasis, chemoresistance, and reduced survival. LncRNAs possess crucial clinical and transitional implications in PCa, as diagnostic and prognostic biomarkers, as well as medicinal targets. To impede the progression of PCa, it is beneficial to target aberrant long non-coding RNAs using antisense oligonucleotides or small interfering RNAs (siRNAs). This prevents them from transmitting harmful messages. In summary, several precision medicine approaches may be used to rectify dysfunctional lncRNA regulatory circuits, so improving early PCa detection and eventually facilitating the conquest of this lethal disease. Due to their presence in biological fluids and tissues, they may serve as novel biomarkers. Enhancing PCa treatments mitigates resistance to chemotherapy and radiation.

**Supplementary Information:**

The online version contains supplementary material available at 10.1007/s00210-024-03736-x.

## Introduction

Prostate cancer (PCa) is the second most common cancer in males and the fifth leading cause of death worldwide. PCa can be asymptomatic and proceed slowly, requiring minimal monitoring. Age greatly affects PCa incidence and death rates, with the highest occurrence in men over 65 (Rawla 2019). Considerations of clinicopathological variables, such as Gleason score, baseline PSA level, patient age, and clinical tumor stage, are crucial to the treatment of localized PCa. High-risk patients (Gleason score > 7, PSA levels > 20 ng/mL, and clinical stage > pT2c) typically benefit from radical prostatectomy or radiotherapy, while those with low-risk disease (Gleason score ≤ 6) are monitored actively. In more than half of the world’s nations, PCa is the most commonly diagnosed cancer in men (112 of 185 nations/territories). Northern and Western Europe, the Caribbean, Australia/New Zealand, North and South America, and Southern Africa have the highest age-standardized incidence rates (Bergengren et al. 2023). PCa is the primary cause of cancer-related deaths among males in 25% of the world’s population (Siegel et al. 2018). Another study revealed that PCa’s overall IR for GOLD and Aurum was 151.7 and 153.1 per 100,000 person-years, respectively, between 2000 and 2021 (Tan et al. 2024).

Androgen hormone, an important steroid, contributes to PCa pathogenesis and masculinity. The androgen-bound androgen receptor (AR) regulates transcription and hormone activity by building homodimers with androgen-responsive elements (AREs) (Heinlein and Chang 2002, Obinata et al. 2017). Radiation and androgen deprivation therapy are the main treatments for locally advanced PCa. Androgen deprivation therapy (ADT) can lower testosterone levels to < 50 ng/mL, causing hormone-refractory tumors. These tumors had high PSA and AR overexpression, indicating castration-resistant prostate cancer (CRPC), a poor prognosis stage (Misawa et al. 2017a). While docetaxel is the standard treatment for CRPC, its effectiveness is limited, and patients experience severe side effects, including anemia and neutropenia (Ritch and Cookson 2016). Thus, better anti-prostate cancer drugs with great efficacy and low toxicity are needed. Natural substances may treat PCa and give novel therapies. Several in vitro and in vivo investigations have shown that natural compounds and extracts may combat PCa (Bai et al. 2021).

Over the past decade, research has shown that non-coding RNAs (ncRNAs) from genomic regions that cannot code for proteins typically change during carcinogenesis. Adverse LncRNA expression contributes to disease development or progression in many human conditions. LncRNA functions include mRNA decoy, alternative splicing, and protein localization modulation. Abnormally expressed lncRNAs can maintain tumor-related signaling pathways or signify early cancer development. Thus, lncRNAs may help diagnose and select PCa treatments. There may be more lncRNAs in PCa, but just a few have been functionally analyzed (Misawa et al. 2017a, Weiss et al. 2014). Additionally, nanoparticles have gained attention for enhancing the effectiveness of chemotherapeutics and radiotherapies by enabling targeted drug delivery and improving the sensitivity of PCa to these treatments (Ashrafizadeh et al. 2024, Mirzaei et al. 2022a).

Recently, phytochemicals have been studied for their effectiveness in suppressing oncogenic LncRNAs and stimulating tumor-suppressive ones. This approach may lower PCa cell growth, proliferation, and metastasis while sensitizing them to conventional therapy (Homayoonfal et al. 2023). This review aims to explore the impact of natural compounds on lncRNA regulation in PCa and assess their ability to overcome chemoresistance.

## LncRNAs: biogenesis and role in PCa

**Fig. 1:**
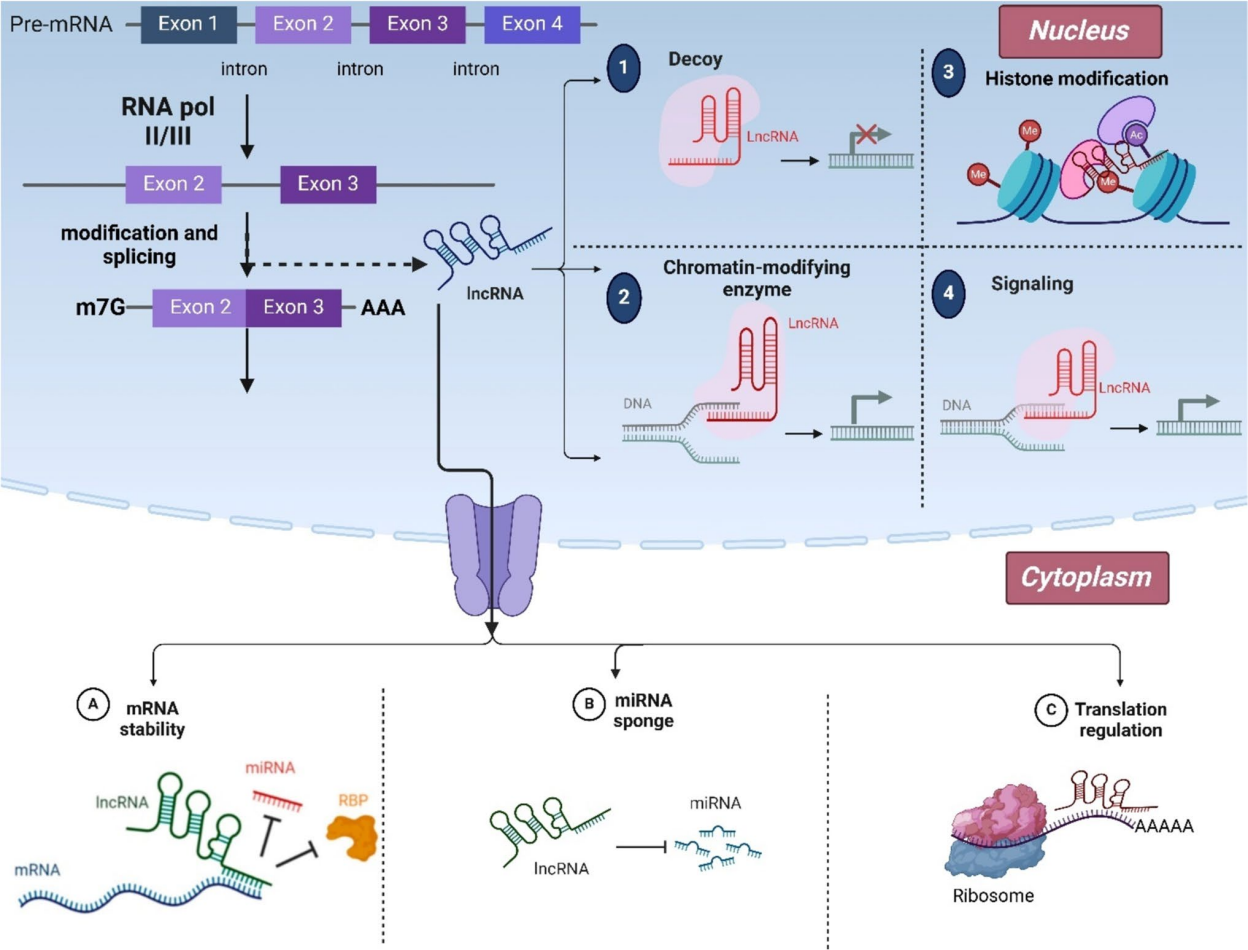
LncRNAs have a crucial role in the alteration of gene expression. LncRNAs exert influence on mRNA stability in various routes such as direct binding to target mRNAs, interactions with RNA-binding proteins, competition with miRNAs as competing endogenous RNAs, regulation of mRNA decay pathways, and interference with transcription

It is estimated that less than 2% of the human genome is made up of genes that encode proteins, while the remaining 98% of the genes are transcribed to RNA without going through the process of encoding proteins. Moreover, non-coding RNAs (ncRNAs) were considered of no particular use in the genome. However, it is now apparent that they play various functional roles within cells. They are categorized based on their size and do not contain long open reading frames. Small ncRNAs such as microRNAs (miRNAs), small interfering RNAs (siRNAs), and PIWI-interacting RNAs (piRNAs) are non-coding transcripts with a length of less than 200 nucleotides. On the other hand, long non-coding RNAs (lncRNAs) are RNA molecules whose length exceeds 200 nucleotides. As of right now, up to 100,000 lncRNAs are known. Different tissues and cancer types express lncRNAs in different ways (Mirzaei et al. 2022a). The lack of an open reading frame (ORF) is the reason lncRNAs cannot encode proteins. Human cancer develops as a result of mutations in noncoding RNAs. RNA polymerase II appears capable of transcribing, capping, polyadenylating, and splicing lncRNAs. Furthermore, the promoter regions, exons, antisense sequences, enhancer sequences, untranslated regions (UTRs) like 3/ and 5/, introns, and intergenic and intragenic regions of the genome can all be used to execute the biogenesis of lncRNAs. Moreover, lncRNAs can regulate target expression through many mechanisms (Fig. 1). LncRNAs can influence biological processes and maintain homeostasis by acting as a signal, decoy, guide, scaffold, and miRNA modulator (Elimam et al. 2024a, c; El-Boghdady et al. 2023).

The position of lncRNAs in the cytoplasm or nucleus of cells determines how they function. Increasing evidence of research indicates that lncRNAs that reside in the nucleus are involved in epigenetic and transcriptional regulation of genes, including DNA methylation, histone modification, chromatin remodeling, interactions with proteins, and transcription factors within the nucleus. Conversely, the lncRNAs that affect the expression of genes at both transcriptional and post-transcriptional levels can be found in the cytoplasm. These cytoplasmic lncRNAs can interact with miRNAs by functioning as competitive endogenous RNAs (ceRNAs), which can impact cytoplasmic proteins and modify RNA metabolism (Fig. 1) (Mirzaei et al. 2022a, Doghish et al. 2024).

Owing to the crucial functions of lncRNAs in cells, they can control the development of tumors, the invasion, and the resistance to medication. According to recent research, lncRNAs are the main regulators of signaling networks in cancer. lncRNAs typically impact miRNAs in malignancies, whereby modulating miRNA expression, lncRNAs impact tumor cell migration and survival (Mirzaei et al. 2022a). Additionally, lncRNAs that promote tumors, like CCAT2, can stop cancer cells from apoptosis (Gao et al. 2020a). Significantly, to support anti-tumor immunity against tumor cells, lncRNAs can encourage the infiltration of immune cells such as B cells, T cells (including CD8 + and CD4 + T cells), neutrophils, and dendritic cells (Zhang et al. 2020).

### Role of lncRNAs in EMT, invasion, and metastasis of PCa

The EMT mechanistic involves the conversion of normal epithelial cells to mesenchymal cells which proliferate easily (Nakazawa et al. 2017). During this process, the epithelial cell markers are suppressed, along with the enhancement of mesenchymal markers, leading to the encouragement of invasion and proliferation (Odero-Marah et al. 2018; Elimam et al. 2024b). Various lncRNAs could modulate EMT by targeting EMT-linked transcriptional factors (Heery et al. 2017). LncRNAs could also act as PRC2 modulators which exhibit a significant role in silencing some genes (Margueron and Reinberg 2011, Simon and Kingston 2009). Recent studies have also indicated that several signaling pathways including AR signaling, STAT3 signaling, and others are involved in inducing and maintaining EMT (Shen et al. 2022). Hence, we attempt to summarize the current knowledge about EMT-related lncRNAs as well as those associated with development, progression, and metastatic capability in PCa.

One of the lncRNAs that modulate the EMT progression is MALAT-1. This lncRNA targets the miR-145, thus promoting PCa development. It has also been shown that MALAT1 modulates the downstream axis SMAD3/TGFBR2 (Zhang et al. 2021) (Table 1). In CRPC, MALAT1 was overexpressed, interacting with EZH2. It also enhanced the invasive and migratory capabilities of PCa cells via repressing target genes such as DAB2IP and BRACHYURY (Wang et al. 2015). Lu et al. disclosed that MALAT1 underscores the beneficial action of the natural product quercetin flavonoid. Quercetin inhibited cancer cell development and tumor growth, suppressed the EMT process, and promoted apoptosis through the downregulation of MALAT1 expression (Lu 2020) (Table 1). A study assessing MALAT1 expression in urinary samples revealed that it was markedly elevated in PCa, suggesting that urinary MALAT1 could serve as a diagnostic marker for PCa (Wang et al. 2014a). CCAT2 is another lncRNA that was markedly elevated in PCa and contributes to the development of PCa. Conversely, silencing CCAT2 reduces PCa progression. Additionally, CCAT2 is implicated in promoting EMT via targeting N-cadherin, vimentin, and E-cadherin (Zheng et al. 2016) (Table 1).

In the same context, DANCR is another PCa modulator and is correlated with advanced stages (Jia et al. 2016). High levels of lncRNA-ATB were reported as PCa promoters via stimulating EMT. Conversely, its suppression alleviates to a great extent the proliferation, invasion, and EMT.

Additionally, overexpression of lncRNA-ATB promoted, and knockdown of lncRNA-ATB inhibited the growth of prostate cancer cells via regulations of cell cycle regulatory protein expression levels. In addition, lncRNA-ATB stimulated EMT associated with ZEB1 and ZNF217 expression levels via ERK and PI3K/AKT signaling pathways (Xu et al. 2016). ZEB1 functions primarily as a transcriptional repressor of epithelial markers such as E-cadherin, while simultaneously promoting the expression of mesenchymal markers like vimentin and N-cadherin. This dual action is essential for the induction of EMT, which is often associated with aggressive tumor behavior and treatment resistance in various cancers. ZEB1 influences epigenetic modifications, such as DNA methylation and histone modifications, which further stabilize the EMT phenotype. For instance, ZEB1 can recruit chromatin-modifying enzymes like histone deacetylases (HDACs) and DNA methyltransferases (DNMTs) to maintain the repression of epithelial genes (Lindner et al. 2020, Wu et al. 2020a) (Table 1).

Another carcinogenic entity is PlncRNA-1, first recognized in PCa, where it regulates the cell cycle and Cyclin D1. As well, PlncRNA-1 encourages EMT by influencing the TGF-β1 axis (Jin et al. 2017, Mirzaei et al. 2022b). Moreover, PlncRNA-1 is nominated as a molecular inhibitor, safeguarding the AR from being inhibited by miRNA-34c and miRNA-297 in PCa (Fang et al. 2016) (Table 1).

UCA1 is also highly expressed in PCa tissue and linked with bad outcomes (Wang et al. 2017a). Its expression was directly associated with Gleason scores, tumor aggressiveness, and bad prognosis. One study revealed a correlation between KLF4 expression in PCa tissue and UCA1 levels, highlighting that the suppression of UCA1 resulted in low levels of KLF4 (Na et al. 2015). KLF4 exerts a critical function in cellular proliferation in metastasis Additionally, UCA1 is reported to function as a ceRNA, downregulating miRNA-204 (Su et al. 2017a). MiR-204 inversely regulates the Sirt1 gene, with overexpression of UCA1 leading to increased Sirt1 levels. Indeed high levels of Sirt1 are linked with cancer cell invasion, migration, and EMT (Wang et al. 2016) (Table 1). Another study disclosed that UCA1 promotes PCa via suppressing miRNA-143 (Yu et al. 2020).

In parallel, ZEB1-AS1 promotes PCa development by targeting ZEB1. Worthy noted that ZEB1 facilitates various tumor biological processes, such as EMT. The upregulation of ZEB1-AS1 modulates the transcription of BMI1 via inhibiting miRNA-200c (Su et al. 2017b). SChLAP1 is shown to characterize an aggressive case of PCa. It opposes the SWI/SNF chromatin-modifying complex, which possesses tumor-preventing role. The SChLAP1 orchestrates tumor cell invasiveness as well as is substantially linked to unfavorable clinicopathological features and bad prognosis (Prensner et al. 2013, Kidd et al. 2021) (Table 1).

HOTAIR is also upregulated in PCa and promotes its proliferation, while its inhibition hinders tumor progression (van der Laak et al. 2021). HOTAIR exerts an oncogenic effect via influencing EZH2, hepaCAM, and MAPK cascade (Battistelli et al. 2017). HOTAIR acts as a scaffold to recruit PRC2 to specific genomic loci. The complex includes EZH2, which is responsible for catalyzing the trimethylation of H3K27 (H3K27me3). Moreover, HOTAIR mediates a switch from histone acetylation to methylation. Specifically, it promotes the conversion of H3K27 acetylation (a marker of active transcription) to H3K27 methylation (a marker of repression). This switch inhibits the transcription of genes like E-cadherin, thus facilitating processes such as EMT in cancer cells (Song et al. 2019, Wasson et al. 2020). Besides, HOTAIR performs its effects via modulating miRNA-34a and miRNA-568 (Wu et al. 2015, Liu et al. 2015a). The lncRNA PCA3 is implicated in the EMT of PCa, where it promotes EMT-promoting genes and abolishes genes that antagonize EMT. Furthermore, PCA3 suppression has been shown to inhibit AR signaling, as well as cell growth and viability (Lemos et al. 2016).

Preceding reports indicated that PCA3 performs its oncogenic role via suppressing miR-1261 which targets the protein kinase D3 gene (Iwasaki et al. 2016). Over and beyond, the lncRNA PCMF1 is involved in the metastasis process in PCa. Cui and his colleagues disclosed that stimulates EMT via modulating miRNA-137. On the flip side, suppressing PCMF1 reversed this effect, restoring miRNA-137’s ability to inhibit the Twist1 protein (Cui et al. 2023) (Table 1). In tandem, the LINC01296 encourages EMT and metastasis in PCa via modulating the PI3K-AKT cascade. Elevation of LINC01296 was markedly associated with unfavorable clinicopathological features and bad prognosis (Wu et al. 2017). Another study reported that LINC01006 advocated PCa development by inhibiting the miRNA-34a-5p (Ma et al. 2020).

**Table 1:** LncRNAs implicated in PCa through the regulation of EMT, invasion, and metastasis

LncRNA	Alteration	Molecular mechanism in PCa	Validation methods	Ref
MALAT-1	Upregulated	Modulates MiR-145-5p-SMAD3/TGFBR2 axis. Interacts with EZH2 and represses PRC2-dependent target genes Promotes EMT	qRT-PCR	(Zhang et al. 2021, Wang et al. 2015)
CCAT2		Knockdown of CCAT2 stimulated EMT through abrogating N-cadherin, vimentin expression, and intensifying the expression levels of E-cadherin	qRT-PCR	(Zheng et al. 2016)
LncRNA-ATB		Enhances ZEB1 and ZNF217 expression via ERK and PI3K/AKT signaling pathways	qRT-PCR	(Xu et al. 2016)
PlncRNA1		GF-β1, N-cadherin, and Cyclin-D1 were downregulated and E-cadherin was upregulated in LNCAP cells after silencing of PlncRNA-1. Moreover, PlncRNA1 sponges miR-34c and miRNA-297	qRT-PCR	(Jin et al. 2017, Fang et al. 2016)
UCA1		Downregulates miRNA-204 and upregulates Sirt1	qRT-PCR	(Su 2017b, Battistelli et al. 2017)
ZEB1-AS1		Depresses miRNA-200c and modulates its target gene BMI1	qRT-PCR	(Su et al. 2017a)
SChLAP1		*SChLAP1* antagonizes the genome-wide localization and regulatory functions of the SWI/SNF chromatin-modifying complex	qRT-PCR	(Prensner et al. 2013)
PCA3		Sponges miR-1261 which negatively regulates PRKD3	qRT-PCR	(Lemos et al. 2016, Iwasaki et al. 2016)
PCMF1		Diminishes the tumor-suppressive effects of miR-137	qRT-PCR	(Cui et al. 2023)
LINC01296		Sponges miR-34a-5p to up-regulate DAAM1	qRT-PCR	(Wu et al. 2017; Ma et al. 2020)
H19	Downregulated	Includes miRNA-675 that represses metastasis	qRT-PCR	(Cai and Cullen 2007)

*EZH2* enhancer of zeste homolog 2, *PRC-2* plybomb repressive complex 2, *ZEB1* zinc finger E-box binding homeobox 1, *ZNF217* zinc finger protein 217, *PRKD3* serine/threonine-protein kinase D3, *DAAM1* disheveled-associated activator of morphogenesis 1

### Role of lncRNAs in PCa progression (apoptosis, proliferation, and tumor growth)

**Fig. 2:**
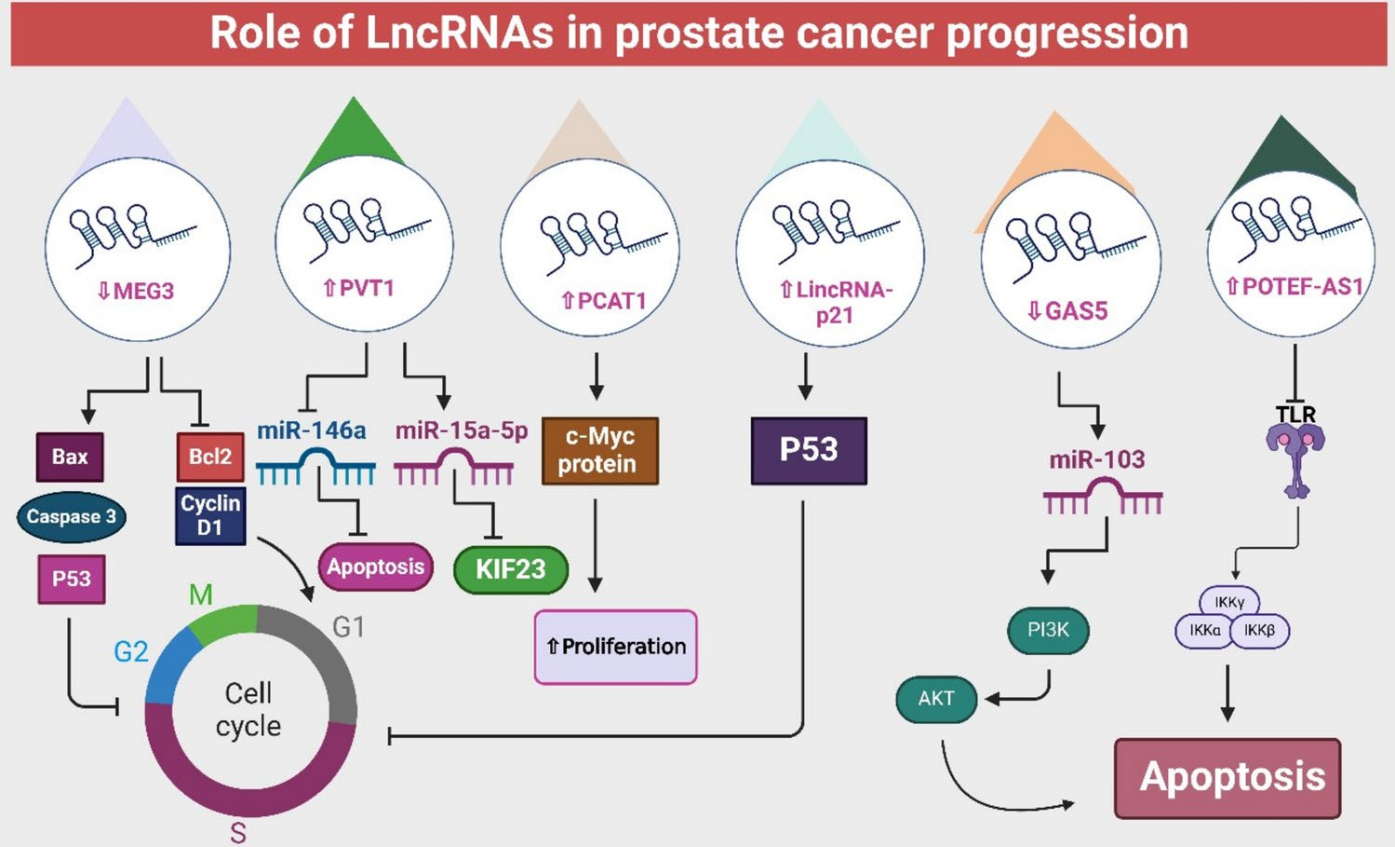
Role of lncRNAs in the progression of PCa including apoptosis, proliferation, and tumor growth. LncRNAs affect key cellular pathways, by interacting with genes and signaling networks, promoting tumorigenesis and disease progression. KIF23: Kinesin-like protein; IKKα: I Kappa B Kinase Alpha; TLR: Toll-like receptors

Numerous researches have demonstrated that lncRNAs perform critical regulatory roles in tumor growth. For instance, GAS5 is downregulated in CRPC, increasing apoptosis and decreasing cell survival in vitro via modulating PI3K-AKT cascade and the miRNA103 (Pickard et al. 2013). The lncRNA SOCS2-AS1 is markedly upregulated in PCa and abrogates cell death (Misawa et al. 2016). Diverse organs are enriched with the lncRNA POTEF-AS1,however, it stimulates PCa development. POTEF-AS1 enhances tumor cell development and hinders apoptosis via TLR and apoptosis cascades (Misawa et al. 2017b) (Fig. 2) (Table 2).

NEAT1 is markedly elevated in PCa samples, suggesting its oncogenic properties (Nitusca et al. 2021). NEAT1 is implicated in diverse oncogenic mechanisms such as invasion, and migration (Fu et al. 2016) (Table 2). MEG3 (Maternally Expressed 3) expression levels were downregulated in PCa (Luo et al. 2015). In vitro studies demonstrated that MEG3 inhibited cell growth and triggered cell death. It was shown to enhance the expression of the pro-apoptotic protein Bax and activate caspase3 while repressing Bcl-2 and Cyclin D1, both of which are linked to cell survival and proliferation. Additionally, MEG3 performs its antitumor effects via activation of p53, which leads to decreased cell proliferation and promotes apoptotic processes (Zhou et al. 2007) (Table 2).

PVT1 is markedly elevated in PCa and plays a potential role in regulating tumor growth and apoptosis via modulating miR-146a (Liu et al. 2016). Moreover, another study showed that the suppression of PVT1 hindered PCa progression by suppressing KIF23 through the stimulation of miRNA-15a-5p (Wu et al. 2020b) (Fig. 2). The oncogenic SPRY4-IT1 is markedly raised in PCa and activates cancer cells’ growth and development. On the other side, suppression of SPRY4-IT1 using siRNA resulted in reduced cellular proliferation and invasion in PC3 cells, along with an increase in apoptosis (Lee et al. 2014, Misawa et al. 2017a) (Table 2). PCAT1 was first identified in PCa but has since shown potential as a biomarker for various cancer types (Prensner et al. 2014a, Liu et al. 2015b, Shi et al. 2015). A transcriptome sequencing study focused on PCa identified 121 unannotated non-coding RNAs in PCa (Prensner et al. 2011). Other investigations have demonstrated that PCAT-1’s role in promoting PCa cell proliferation relies on the modulation of the c-Myc protein (Prensner et al. 2014b) (Table 2).

LincRNA-p21 was recorded as a PCa suppressor where it attenuates growth and colonization of PCa. Furthermore, it triggered cell death and modulated p53. Remarkably, suppressed LincRNA-p21 is linked with aggressive clinical features and bad prognosis (Wang et al. 2017b).

Intriguingly, AR-regulated lncRNAs, CRPC LncRNAs, are elevated in CRPC. Suppression of lncRNAs PRKAG2AS1 and HOXCAS1) lessened CRPC tumor growth, showing repression of AR. Precisely, subcellular localization of the splicing factor, U2AF2, with an essential role in AR splicing machinery was modified according to the level of HOXCAS1 (Takayama et al. 2020) (Fig. 2).

As mentioned in the current review, there is great progress and knowledge about the interplay of lncRNAs in proliferation, invasion, apoptosis, EMT, and metastasis in PCa; however, still diverse gaps remain. The manner through which lncRNAs interact with multiple protein targets and transcription factors to regulate genes involved in proliferation, invasion, and migration is not fully elucidated. The accurate underlying mechanisms through which these lncRNAs perform their role in EMT and metastasis as well as affect the integrity of the epithelial cell junctions are not fully investigated. Moreover, some lncRNAs mediate their roles via interactions with chromatin modifiers, transcription factors, and modulating miRNAs, but detailed mechanistic insights are still lacking and need further mechanistic studies. Another key point that should be taken into consideration is the heterogeneity of the PCa; thus, the regulation of the lncRNAs is variable according to the tumor subtypes, malignant stages, and their molecular features.

TRPM2-AS is elevated in PCa and associated with bad outcomes. In vitro research revealed that its silencing leads to the enhancement of apoptosis. Importantly, TRPM2-AS was disclosed as a potential coordinator of diverse genes related to survival, and the cell cycle (Orfanelli et al. 2015), (Lavorgna et al. 2015) (Table 2).

**Table 2:** LncRNAs implicated in PCa progression

LncRNA	Alteration	Molecular mechanism in PCa	Validation method	Ref
GAS5	Downregulated	GAS5 interacted with E2F1 and enhanced the binding of E2F1 to the P27^Kip1^ promoter	qRT-PCR	(Luo et al. 2017)
MEG3		Enhancement of Bax expression and activation of caspase-3, while repressing Bcl-2 level and Cyclin D1. Activation of p53	qRT-PCR	(Zhou et al. 2007)
SOCS2-AS1	Upregulated	Modulating the epigenetic control for TNFSF10 and thereby repressing apoptosis	Microarray analysis qRT-PCR	(Misawa et al. 2016)
POTEF-AS1		*POTEF-AS1* promoted cell growth, repressed genes related to the Toll-like receptor signaling and apoptosis pathways, and inhibited apoptosis in docetaxel-treated LNCaP cells	RNA Seq qRT-PCR	(Misawa et al. 2017b)
NEAT1		Its oncogenic role is highly dependent on the NEAT1-CDC5L-AGRN circuit	qRT-PCR	(Li et al. 2018)
PVT1		Regulates the expression of miR-146a. Also acts by suppressing KIF23 through the stimulation of miR-15a-5p	qRT-PCR	(Liu et al. 2016, Orfanelli et al. 2015)
SPRY4-IT1		Its knockdown using siRNA resulted in reduced cellular proliferation and increased apoptosis		(Lee et al. 2014, Misawa et al. 2017a)
PCAT1		Regulates the c-Myc protein. Functions as a sponge for miR-145-5p modulates the expression of FSCN1	qRT-PCR	(Prensner et al. 2014b, Xu et al. 2017)
LincRNA-p21		Improves the expression of p53-responsive genes by modulating their binding to promoter regions	qRT-PCR	(Wang et al. 2017b)
TRPM2-AS		Coordinating the expression of numerous genes of survival, unfolded protein response, and cell cycle	microarray analysis qRT-PCR	(Orfanelli et al. 2015, Lavorgna et al. 2015)

*TNFSF10* tumor necrosis factor ligand superfamily member 10, *CDC5L* cell division cycle 5-like protein, *FSCN1* fascin actin-bundling protein 1

### Role of chemotherapy in the treatment of PCa

Docetaxel-based chemotherapy initially demonstrated a slight increase in survival when compared to mitoxantrone-based therapy as first-line chemotherapy in 2004; therefore, chemotherapy has been essential in the treatment of metastatic CRPC (mCRPC) (Antonarakis and Armstrong 2011). The antimitotic drug docetaxel has been shown in recent years to suppress androgen receptor (AR) transcriptional activity and its longstanding ability to prevent microtubule disassembly. Docetaxel can prevent the AR from moving to the nucleus in response to ligand-dependent signaling pathways as well as androgens. By influencing the gene promoter, docetaxel also suppresses the expression of the AR gene. It raises the amounts of Forkhead box O1 (FOXO1), a strong transcriptional repressor of AR (Fitzpatrick and de Wit 2014). Abiraterone acetate and enzalutamide are two new oral antiandrogen medications that have increased survival in pre- and post-docetaxel situations since the approval of docetaxel (Ryan et al. 2013, Beer et al. 2014). Furthermore, post-docetaxel patients’ lives have been prolonged with cabazitaxel, a second and stronger taxane (De Bono et al. 2010).

Both docetaxel and paclitaxel are recommended as well-known drugs for the chemotherapy of prostate tumors. They also serve a similar purpose in cancer treatment, which is focused on preventing microtubule depolymerization, disrupting microtubule equilibrium, and so delaying the advancement of the cell cycle (Hashemi et al. 2023). Recent evidence suggests that Doxorobucin has pleiotropic anticancer properties, including its participation in immunomodulation, apoptosis, senescence, autophagy, ferroptosis, pyroptosis induction, DNA damage, and the generation of reactive oxygen species (ROS) (Kciuk et al. 2023). It was concluded that Doxorubicin increases the cytotoxicity and apoptosis caused by TRAIL targeting prostate cancer cells (Wu et al. 2002; El-Hawwary et al. 2022; Bakr et al.^2021^).

### Therapy resistance to the major treatments in PCa

The PCa poses a significant health challenge due to its resistance to radiation therapy and chemotherapy. Patients with metastatic PCa frequently have a dismal prognosis, even with rigorous therapies utilizing a variety of techniques, while those with localized PCa typically have a better survival rate (Sarfraz et al. 2024). In addressing PCa, chemoresistance (CR) and radioresistance (RR) present major problems. CR represents a major hurdle in PCa management. Even though CT is a common therapeutic strategy for PCa, the development of CT resistance is a considerable clinical barrier. A comprehensive understanding of the underlying mechanics is crucial for the efficient treatment of PCa (Bhagirath and Saini 2019).

There is growing evidence that lncRNAs have a significant role in carcinogenesis, specifically in PCa. Furthermore, lncRNAs play a significant role in treatment resistance in PCa (Sarfraz et al. 2024), progression and CR being significantly overexpressed in CRPC cells. Silencing of HOXD-AS1 reduced cell proliferation in vitro and inhibited tumor progression in vivo in PCa by triggering the cell cycle arrest transition from G2 to M phase (Gu et al. 2017) (Table 3). Wang et al. (2021) demonstrated that in docetaxel-resistant PCa cells and tissues, the lncRNA OGFRP1 was highly expressed, additionally,it was possible to enhance these cells’ susceptibility to docetaxel and paclitaxel both in vitro and in vivo by suppressing OGFRP1 via the upregulation of miR149-5p and the subsequent downregulation of IL-6. Functionally, OGFRP1 was capable of binding to miR-149-5p and encouraging the overexpression of IL-6, an essential modulator of cancerous activity both in vivo and in vitro (Wang et al. 2021) (Table 3).

CCAT1 is another oncogenic lncRNA implicated in paclitaxel resistance in PCa; a study by Li et al. (2020) demonstrated that knocking out CCAT1 prevents the development, migration, and expansion in PCa cells. Sponging of miR-24–3p by CCAT1 hinders it from the activation of its downstream protein (FSCN1) as a consequence of this association; FSCN1 is highly expressed which increases paclitaxel tolerance (Li et al. 2020) (Table 3).

**Fig. 3:**
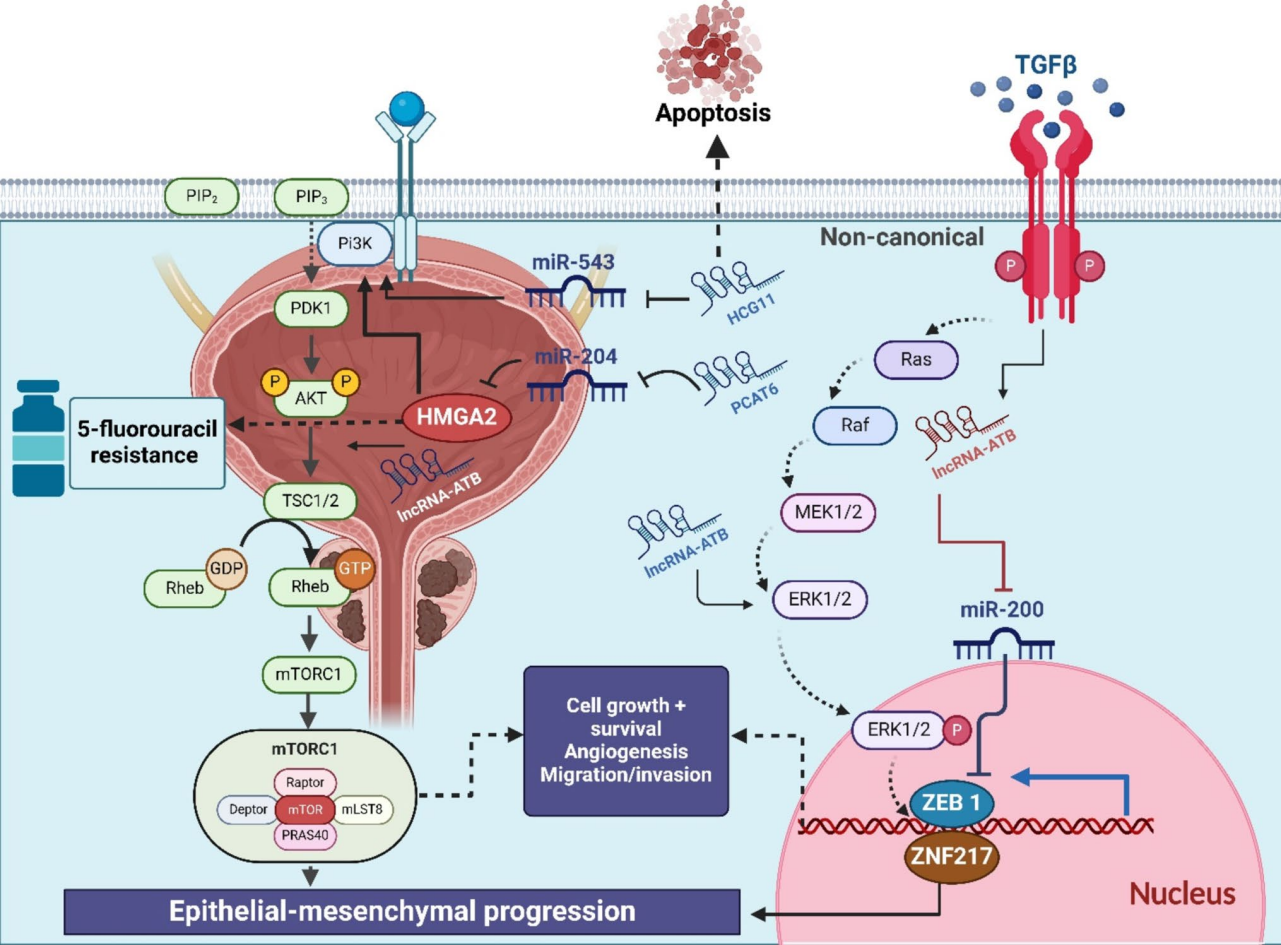
The role of the PI3K/Akt/mTOR pathway in chemotherapy resistance and EMT in PCa. Dysregulated long non-coding RNAs (lncRNAs), such as PCAT6, HCG11, and lncRNA-ATB, modulate this pathway by interacting with miRNAs (e.g., miR-204, miR-543) and downstream targets (e.g., HMGA2, ZEB1). These interactions drive resistance to 5-fluorouracil (5-FU) and enhance epithelial-to-mesenchymal transition (EMT), influencing PCa progression and therapeutic response. Ras: Rat sarcoma gene; Raf: rapidly accelerated fibrosarcoma; MEK1/2: mitogen-activated protein kinase; ERK: extracellular signal-regulated kinase; ZEB1: zinc finger E-box binding homeobox 1; ZNF217: zinc finger protein 217; PIP3: phosphatidylinositol (3,4,5)-trisphosphate; PDK1: 3-phosphoinositide-dependent kinase 1; AKT: alpha serine/threonine-protein kinase; TSC1/2: tuberous sclerosis proteins 1 and 2; Rheb: Ras homolog enriched in brain; mammalian target of rapamycin complex 1

Moving to another oncogenic lncRNA; HOTTIP which is highly expressed in PCa and associated with CR. Jiang et al. (2019) demonstrated that HOTTIP suppression inhibited the Wnt/β-catenin pathway leading to reduced CDK4, cyclin D1, and β-catenin expressions; consequently, the development of PCa cell and cell cycle will be inhibited; moreover, the sensitivity of the cells to cisplatin will be enhanced (Jiang et al. 2019) (Table 3). MALAT-1 was significantly expressed in cell lines and PCa tissues (Ren et al. 2013, Dai et al. 2019). Additionally, its overexpression with serine/arginine-rich splicing factor 1 (SF2) upregulation promoted the production of androgen receptor splicing variant 7 (AR-v7) which improved the Enzalutamide resistance of PCa cells (Wang et al. 2017) (Table 3). Wang et al. (2016) explained that miR-204 negatively modulated Sirt1 expression in prostate cancer cells. UCA1 upregulation directly resulted in decreased miR-204 expression. UCA1 overexpression resulted in increased Sirt1 expression in PNT2 cells, while knockdown of endogenous UCA1 led to decreased Sirt1 in LNCaP and 22RV1 cells. UCA1 siRNA, Sirt1 siRNA, and miR-204 mimics could enhance docetaxel-induced activation of caspase-3 and cell apoptosis in 22RV1/DR cells (Wang et al. 2016). Another study revealed that lncRNA NEAT1 exerts oncogenic function in PCa by competitively “sponging” both miR-34a-5p and miR-204-5p. Inhibition of miR-34a-5p or miR-204-5p expression mimics the docetaxel-resistant activity of NEAT1, whereas ectopic expression of miR-34a-5p or miR-204-5p attenuates the anti-drug function of NEAT1 in PCa cells. Besides, ACSL4 was found to be a target of both miR-34a-5p and miR-204-5p, and ACSL4 was also inhibited by miR-34a-5p and miR-204-5p. Moreover, suppression of miR-34a-5p or/and miR-204-5p greatly restrained the expression of ACSL4 upon NEAT1 overexpression. Joint expression of miR-34a-5p and miR-204a-5p synergistically decreased the expression of ASCL4, indicating miR-34a-5p and miR-204a-5p collaboratively inhibit the expression of ACSL4 (Jiang et al. 2020) (Table 3). One of the significant hurdles in PCa treatment is the resistance of cancer cells to chemotherapeutic agents, including 5-FU. Studies reveal that the activation of the PI3K/Akt/mTOR pathway plays a pivotal role in this resistance. By promoting cell survival and reducing susceptibility to apoptotic signals (Wang et al. 2019), this pathway enables cancer cells to endure even the most potent therapeutic regimens. For instance, lncRNA PCAT6 has been implicated in chemotherapy resistance. PCAT6 activates the HMGA2/PI3K axis by suppressing miR-204, which would otherwise inhibit PI3K signaling. This dysregulation results in heightened resistance to 5-FU, a common chemotherapeutic agent used in PCa treatment (Wu et al. 2019). On the other hand, certain lncRNAs exhibit tumor-suppressive roles by disrupting this pathway. LncRNA HCG11, for example, hinders PCa progression by downregulating miR-543, a microRNA that promotes PI3K/Akt signaling. By blocking this oncogenic cascade, HCG11 induces apoptosis, a process that is otherwise suppressed in advanced PCa. These findings highlight the dual role of lncRNAs, acting either as oncogenes or as tumor suppressors, within the context of the PI3K/Akt/mTOR pathway (Fig. 3).

Considering efficient therapy for patients with radioresistant tumors needs larger doses of radiation, which will have adverse effects; therefore, finding markers of radiosensitivity and variables contributing to RR is essential in determining responsive patients and enhancing the effectiveness of radiation therapy (Podralska et al. 2020). One of the molecular mechanisms implicated in the RR of PCa includes autophagy surviving cell process that helps tumor cells resist therapeutic stresses. Radiation-induced autophagy allows for the degradation and recycling of damaged cellular components, hence maintaining cellular homeostasis,thus, the process is often upregulated in radiation-treated PCa cells and contributes to their survival (Ahmadi-Dehlaghi et al. 2023). For example, it is known that radiation suppresses the mTOR signaling pathway, which is one of the major negative regulators of autophagy,hence, the induction of autophagy confers resistance to cell death (Sun et al. 2021). Some studies have also pointed out that lncRNAs, like HULC, are bound with Beclin-1, preventing its phosphorylation and promoting autophagy upon radiation stress. Knockdown of HULC has promoted autophagy, suppressed the mTOR cascade, and increased cell death post-radiation (Chen et al. 2018). Additionally, Xiu et al. (2020) found that the radiosensitivity of PCa was increased upon TUG1 and SMC1A decreased levels via modulating miR-139-5p,therefore, the combination of TUG1 knockdown with radiation may serve as a potential therapeutic target for PCa (Xiu et al. 2020) (Table 3).

LncRNAs have significance in PCa RR; multiple studies have discovered different lncRNAs (Sarfraz et al. 2024). Jiang et al. (2019) investigated how GAS5 influences miR-18a to increase α-Solanine-induced radiosensitivity in human PCa cells decreasing cell viability and survival and inducing apoptosis. Their findings suggest that GAS5 overexpression and miR-18a suppression may be used as a treatment approach for PCa (Jiang et al. 2019).

As prevailed in the current review, the role of lncRNAs in the development of drug or radiation resistance is an emerging area of research. However, there are some uncovered issues such as the absence of a comprehensive mechanistic of the responsible signaling pathways implicated in the development of this resistance. Although it is known that many lncRNAs interacted with many physiological processes such as DNA repairing, and programmed cell death, it is yet unexplained how these interactions predispose to chemotherapy resistance in PCa. To counteract the developed therapeutic resistances, deeper studies are needed to determine how lncRNAs interact precisely with signaling pathways that govern these resistances.

**Table 3:** Resistance to the major treatments in PCa

LncRNAs	Expression profiling in PCa	Mechanism of action in drug/radioresistance	Ref
HOXD-AS1	Upregulated	Through the modulation of cell cycle phases, cell proliferation, and tumor progression were inhibited upon HOXD-AS1 suppression	(Gu et al. 2017)
OGFRP1		It enhances chemoresistance via sponging miR-149-5p, promoting IL-6 expression in vitro and in vivo	(Wang et al. 2021)
CCAT1		It served as miR-24–3p’s sponge and it positively controlled FSCN1	(Li et al. 2020)
HOTTIP		It activated the Wnt/β-catenin signaling pathway and its downstream mediators	(Jiang et al. 2019)
MALAT-1		It upregulated SF2/AR-v7 axis to enhance CR	(Wang et al. 2017)
UCA1		Increased levels of UCA1 and Sirt1 with decreased levels of miR-204 enhanced docetaxel resistance	(Wang et al. 2016)
NEAT1		Inhibition of miR-34a-5p and miR-204a-5p significantly reduced ACSL4 following the overexpression of NEAT1	(Jiang et al. 2020)
HULC		Radioresistance is increased via autophagy inhibition and mTOR activation	(Chen et al. 2018)
TUG1		It modulates miR-139-5p	(Xiu et al. 2020)
GAS5	Downregulated	In PCa cells, GAS5 was reduced and miR-18a was elevated; however, these changes reverted upon the availability of α-solanine	(Jiang et al. 2019)

*FSCN1* fascin actin-bundling protein 1, *SF2* splicing factor 2, *Sirt1* sirtuin, *ACSL4* acyl-CoA synthetase long-chain family member 4, *mTOR* mammalian target of rapamycin

### Targeting lncRNA in PCa treatments by natural products

Despite the promising results from preclinical studies, clinical applications of lncRNA-targeting strategies using natural products are still limited. More research is needed to understand the precise roles of various lncRNAs in prostate cancer and how natural compounds can be effectively utilized in clinical settings. The integration of lncRNA profiling into therapeutic strategies could enhance personalized medicine approaches for prostate cancer patients. For example, studies have shown that quercetin can inhibit the growth of various cancer cell lines, such as breast, colon, and prostate cancer. These effects are partially mediated through the modulation of lncRNAs like MALAT1, which influences cell proliferation and metastasis. Genistein inhibits the expression of oncogenic lncRNAs such as HOTAIR and MALAT1 in various cancer cell lines. In mouse models, the administration of genistein reduces tumor growth by downregulating these lncRNAs. Clinical studies on quercetin in cancer therapy are still limited but show promise. Some trials have reported its use as an adjunct to traditional therapies, particularly in combination with chemotherapy, where quercetin may alter lncRNA profiles associated with CR.

### Genistein

Genistein is extensively available in numerous legumes including cauliflower, broccoli, sunflower, and clover seeds (Mei et al. 2019). It has been demonstrated that genistein inhibits PCa via the down-regulation of lncRNA HOTAIR and the elevation of miR-34a level in PC3 and DU145. These effects lead to enhanced apoptosis, cell arrest, and inhibition of proliferation, invasion, and migration (Chiyomaru et al. 2013). A similar effect was observed in a study by Chen et al. (Chen et al. 2015) on MCF-7 cells, where genistein reduced Akt phosphorylation and HOTAIR expression (Chen et al. 2015). Another study revealed that genistein exerts its anti-cancer activity by decreasing the expression of lncRNA TTTTY18 and Akt phosphorylation in colorectal cell lines (Chen et al. 2020).

**Fig. 4:**
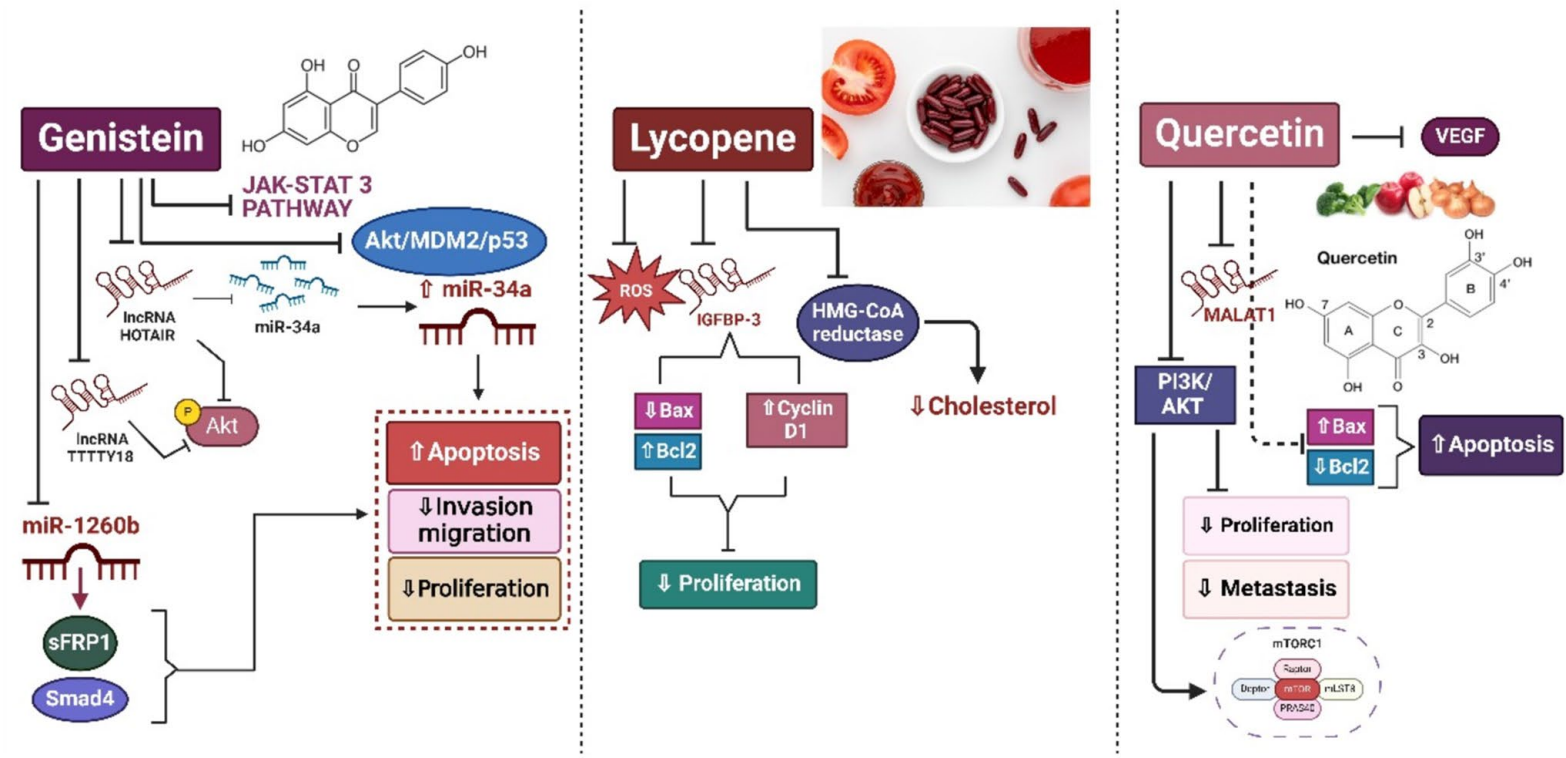
Natural products as a regulator of lncRNA in targeting PCa treatments. This illustration depicts the performance of natural compounds such as genistein, lycopene, and quercetin in influencing lncRNA expression and modulating key pathways involved in PCa progression, proliferation, apoptosis, and metastasis

Genistein modulates the expression of ER*-β* on the ER-*β* promoter methylation process in PCa cell lines reducing cancer cell proliferation by decreasing promoter methylation (Mahmoud et al. 2015). In PCa cell lines, genistein suppresses the expression of miRNA-1260b and its target genes (sFRP1 and Smad4) which leads to the inhibition of cell invasion, migration, and proliferation and stimulates apoptosis (Hirata et al. 2014). In parallel with these results, a study performed by Mahmoud and his colleagues showed that genistein inhibited cancer cell growth in a dose-dependent manner (Mahmoud et al. 2013). In mCRPC cells, it enhances the response to cabazitaxel therapy by inducing apoptotic signals and proapoptotic Bax protein, where the combination of both treatments significantly reduced the growth of mCRPCs in the in vivo model (Zhang et al. 2013). Genistein hindered Akt/MDM2/p53 and JAK/STAT3 signaling pathway via the inhibition of the phosphorylation of JAK1, JAK2, and STAT3 and the inhibition of Akt and MDM2 which prevents the growth of cancerous cells (Gao et al. 2020b) (Fig. 4).

### Lycopene

Tomatoes are the primary source of lycopene, an acyclic carotenoid naturally present in many plants. It is an acyclic carotenoid present naturally in most plants (Song et al. 2021). It has been reported to possess anti-proliferative activity by preventing the progression of the cell cycle in PCa cell lines (Ford et al. 2011). Moreover, lycopene modulates the expression of IGFBP-3 which promotes apoptosis via upregulating *Bax* and downregulating the expression of cyclin D1 and *Bcl-2* in PC-3 cells, preventing cell proliferation (Wang and Zhang 2007) (Fig. 4). Another mechanism for lycopene is that it reduces the total cholesterol levels by inhibiting the expression of HMG-CoA reductase and inhibiting Ras in PC-3 and LNCaP tumor cells. Additionally, it reduces ROS and phosphorylated JNK (Palozza et al. 2010).

### Quercetin

Quercetin is a polyphenol molecule and the most common flavonoid in fruits, vegetables, and medicinal plants, including barks, flowers, leaves, nuts, and seeds. It can also be found in a variety of foods such as apples, berries, grapes, onions, tea, and tomatoes (Wang et al. 2022). Ward et al. (2018) proved that quercetin therapy significantly reduced the cell viability of PCa cells (LNCaP, PC-3, and DU-145) in a time- and dose-dependent manner, indicating that quercetin exerts its anti-cancer activity by regulating Akt, ROS, and NF-κB pathways (Ward et al. 2018, Hatawsh et al. 2024).

Quercetin repressed the expression of the level of oncogenic lncRNA MALAT1 in PC-3 cells, hence impeding their proliferation, and also reduced the PI3K/AKT signaling pathway, accelerated apoptosis, and it inhibited metastasis via the EMT process (Lu et al. 2020). Following quercetin treatment, PC-3 cells exhibited a decline in anti-apoptotic Bcl-2 levels and an increase in pro-apoptotic Bax,additionally, the ER stress-associated proteins were elevated which in turn led to apoptosis (Liu et al. 2014). Another research has demonstrated that the treatment of PC-3 cells with quercetin disrupts the transport of mRNA by hnRNPA1 from the nucleus to cytoplasm leading to the accumulation of hnRNPA1 in the cytoplasm and subsequently translocation to the stress granules which eventually causes cells to undergo apoptosis (Ko et al. 2014). Additionally, apoptosis could be induced by the treatment of PCa cells with quercetin via the PARP cleavage, while blocking the AKT/mTOR/P70S6K cascade and reducing VEGF secretion (Pratheeshkumar et al. 2012) (Fig. 4).

Targeting lncRNAs in PCa treatment using natural products is challenging and needs to be addressed for more effective and targeted therapies. However, it is crucial to dig deeper into understanding the molecular interactions through which natural products cooperate to regulate lncRNAs in an attempt to develop more specific therapies. Moreover, most of the studies on natural products and their modulatory effects on lncRNAs were conducted on cell lines, so it is important to validate these natural products on animal models as a preliminary stage before the clinical investigations. Assessment of the natural products’ specificity and the delivery system is also needed to achieve more effective outcomes.

### Role of lncRNAs in diagnosis and prognosis of PCa

One important method for screening PCa is the prostate-specific antigen (PSA). PSA is specific to the prostate but not to PCa, as its levels depend on several factors. Since PSA levels might be normal even in the presence of PCa, determining PSA levels is not 100% sensitive to PCa. The maximum sensitivity and specificity are obtained with a PSA cutoff value of 4.0 ng/mL. PSA levels in the serum can be measured to identify PCa early on. PSA testing has been linked to high rates of overdiagnosis and overtreatment, but it may reduce the mortality rate from PCa. Some other clinical and imaging methods can be utilized to increase the detection of clinically relevant malignancies (De Visschere et al. 2010, Dejous and Krishnan 2021). PSA analysis has been superseded by the pro-PCa antigen 3 (PCA3) clinical test, which is authorized by the FDA. PCa can be diagnosed more quickly and simply using PCA3 detection than PSA because of its high specificity and sensitivity.

Although PSA investigation is an important tool for managing PCa, its usage in the screen of asymptomatic individuals is considered a complex challenge (Jain et al. 2023). Considering the rapid progress of tumor biology, many lncRNAs have shown crucial roles in the growth and start of malignancies (Aborehab et al. 2023). A variety of innovative methods for analyzing and detecting lncRNAs are currently being explored. One notable advancement is digital droplet polymerase chain reaction (ddPCR), which offers exceptional precision, sensitivity, and the ability to achieve absolute quantification of nucleic acids. In ddPCR, the sample is partitioned into numerous independent PCR sub-reactions, with each partition containing either a few or no target sequences. Following PCR amplification, the fraction of positive partitions is analyzed to determine the concentration of the target sequence using Poisson statistics, yielding results with statistically defined accuracy. This method efficiently concentrates target sequences within isolated microreactors, which minimizes template competition and facilitates the detection of rare mutations in the presence of wild-type sequences. Additionally, ddPCR demonstrates a higher tolerance for inhibitors found in samples and eliminates the need for a standard curve for quantification (Taylor et al. 2017, Quan et al. 2018a). Another powerful technique is RNA fluorescence in situ hybridization (RNA-FISH), which enables detailed examination of spatial patterns and mechanisms of gene expression across various biological scales, from single cells to entire organisms (Santini et al. 2021). Likewise, in situ hybridization (ISH), a method similar to RNA-FISH, allows the localization of lncRNA expression using a labeled probe to complementary sequences in fixed cells or tissues and then visualizing it by chromogenic, fluorescent, or electron microscopic methods (Chu et al. 2019). Also, lncRNA microarrays serve as a robust tool for investigating lncRNAs in both biological and disease contexts in large-scale studies (Shi and Shang 2016).

Current research indicates that aberrant expression of circulating lncRNA in biological bodily fluids such as serum, saliva, and others is important for early diagnosis and pathological grade categorization (Quan 2018b; Hamdy et al. 2024; Ismail et al. 2024; Abd-Elmawla et al. 2023). Yan and his colleagues stated that PCAT11 could be a diagnostic indicator for PCa where low expression of PCAT14 was associated with PCa hallmarks (Yan et al. 2021). Meanwhile, elevated expression of PCAT-1 was linked to the progression of PCa, and this was confirmed via Kaplan–Meier curves and Cox regression analysis,therefore, it might be a useful prognostic biomarker for PCa patients (Li et al. 2020). Another study revealed the clinical usage of MALAT1 in diagnosing PCa with AUC = 0.670 for patients with PSA 4 ng/ml and AUC = 0.742 for patients with PSA 10 ng/ml. These findings are comparable with PCA3 where AUC ranges from 0.64 to 0.69 (Wang et al. 2014a, Auprich et al. 2011). Nevertheless, increased expression levels of MALAT1 are linked to signs of poor prognosis (Ren et al. 2013). LncRNA-p21 levels may help distinguish PCa from benign disease and also be used to discriminate PCa patients with sensitivity = 67% and specificity = 63% (Işın et al. 2015). The lncRNA MAGI2-AS3 is a novel marker studied in various diseases (Kortam et al. 2023). Hu et al. revealed that MAGI2-AS3 could be a diagnostic biomarker in PCa. MAGI2-AS3 exhibited AUC = 0.953 with sensitivity and specificity of 91.5% and 84.7%, respectively (Hu et al. 2022). It has been shown that MAGI2-AS3 acts as a tumor suppressor gene that prevents PCa from proliferating and migrating via various mechanisms. A negative prognosis for PCa could be predicted using downregulated MAGI2-AS3 (Yang et al. 2023). Bayat and his colleagues reported that Prcat17.3 and Prcat38 could be used in differentiating PCa, where their expressions were elevated as well as possess AUC = 0.92 and 0.77, respectively (Bayat et al. 2018).

Similarly, the lncRNA SNHG9 plays a significant role in both the diagnosis and prognosis of PCa, Li et al. (2021) found that the increased level of SNHG9 in PCa was correlated with unsatisfactory clinical outcomes. Furthermore, SNHG9 may influence ribosome and immune infiltrating cell function, which could contribute to PCa development (Li et al. 2021). lncRNA ZEB1-AS1 and UCA1 were significantly expressed in PCa tissues and connected with poor prognosis, in which ZEB1-AS1 has been linked to the metastasis of lymph nodes, distant metastasis, medical stage, and histopathology type, as well as it modulates BMI1 that is correlated to metastasis and unsatisfactory prognosis (Su et al. 2017b). Reduced patients’ overall survival (OS), a greater Gleason score, and an elevated TNM stage were all positively connected with the high expression of UCA1 (Zhang et al. 2017) (Fig. 4). Another study reported that the latter lncRNA could modulate CXCR4 expression levels of CXCR4 in PCa cells via sponging miRNA-204 to promote PCa metastasis, where the poor prognosis was linked with the elevated level of CXCR4 (Chen and Zhong 2015, He et al. 2019).

**Fig. 5:**
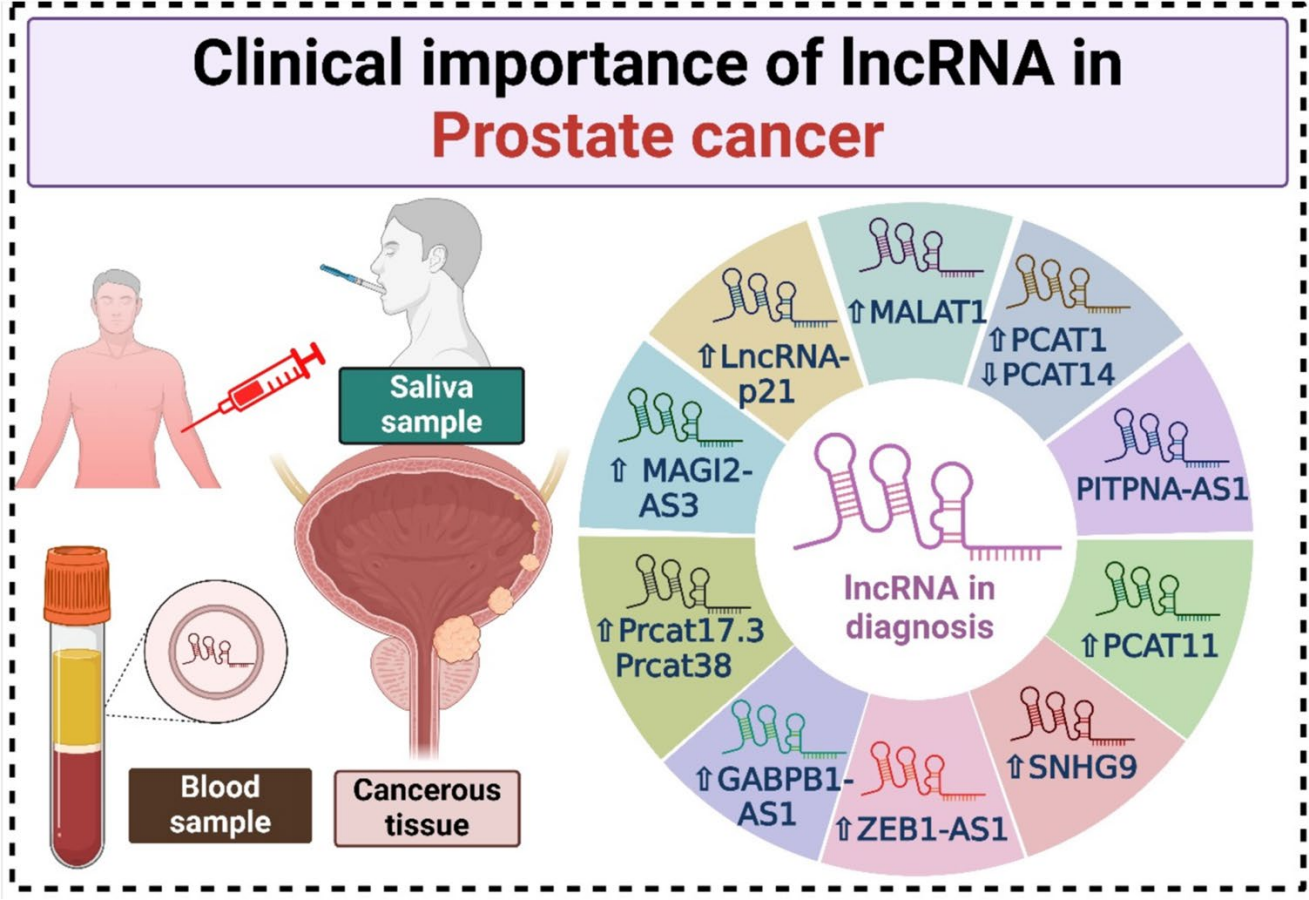
Clinical importance of lncRNAs in PCa. This representation demonstrates the utility of lncRNAs as valuable biomarkers in detecting and diagnosing PCa, as well as their roles as noninvasive approaches for early detection through blood, saliva, and tissue samples

In parallel, Chen et al. stated that GABPB1-AS1 can be used in PCa diagnosis (Chen et al. 2024). The lncRNA PVT1 was significantly increased in PCa tissues compared to normal ones, the AUC = 0.860, which indicated that PCa could be effectively diagnosed by evaluating PVT1 expression, and poor prognosis was observed for those expressing high levels of PVT1 (Liu et al. 2021). Additionally, it was associated with disease progression and the pathological stage where its elevation was linked with a low survival rate (Yang et al. 2017). PITPNA-AS1 also exhibited high sensitivity and specificity scores in diagnosing PCa (Song et al. 2024) (Fig. 5). Nitusca et al. elucidated that NEAT1 exhibits clinical interest in diagnosing PCa where it was markedly high among patients and achieved AUC = 0.72 (Nitusca et al. 2021). Notably, NEAT1 promotes neoplastic growth by altering the epigenetic environment to favor cancer-promoting gene regulation (Chakravarty et al. 2014). Recently, Amirmahani et al. (2024) reported the diagnostic role of CAT2064 and CAT2042 with AUC = 0.87 and 0.84, respectively (Amirmahani et al. 2024). Additionally, the significantly elevated GAS5 was also suggested in PCa diagnosis (Lu et al. 2017). Another two molecular markers were recorded as indicators of PCa which are lncRNAs*TAPIR-1* and *−2*. Relatedly, these two markers were targeting p53 (Friedrich et al. 2020).

Finally, there is a lot of promise in using lncRNAs to diagnose and predict PCa as shown in the current review. In an attempt to validate their utility, standardization and large-scale clinical validation across different tumor subtypes are required to be more reliable and reproducible. All of these ultimately will improve patient outcomes and will aid in establishing more personalized treatment strategies.

Interestingly, lncRNAs often interact with proteins to participate in multiple levels of gene regulation. Therefore, identifying the RNA-binding proteins and validating their interaction with lncRNAs will help uncover the functions of lncRNAs (Ye et al. 2024). Various techniques have been developed to identify these interactions. Of these is cross-linking and immunoprecipitation (CLIP) which enables the identification of RNAs, including lncRNA, that bind with particular proteins of interest. CLIP involves the cross-linking of proteins to target RNAs. UV cross-linking during the CLIP procedure results in the formation of a covalent bond between the RNA and the nearby amino acid moiety and therefore generally used to reveal direct lncRNA–protein interaction in living cells. Following cross-linking, immunoprecipitation of the protein of interest and its associated RNAs with specific antibodies permits further detection of protein-bound RNA fragments (Darnell et al. 2018). Another in vitro method is RNA pull-down assay in which a biotinylated lncRNA is synthesized in vitro and then complexed with either in vitro synthesized proteins to confirm a direct interaction or protein in cell lysates to reveal both direct and indirect binding partners. After that, the biotin–RNA–protein complex is purified using magnetic beads and detected by mass spectrometry or Western blot (Darnell et al. 2018).

### Post-transcriptional regulation of lncRNAs in PCa

The intricate post-transcriptional regulation of lncRNAs is essential to understanding their involvement in PCa initiation, progression, and therapy resistance. One crucial mechanism that increases the diversity of lncRNA transcripts is alternative splicing. This mechanism can produce different isoforms of a single lncRNA gene in prostate tumors, each of which may have unique regulatory functions or activities (Liu et al. 2021). For example, the prostate cancer antigen 3 (PCA3) lncRNA has been extensively studied. Prostate tissues reportedly contain at least four splice variants of PCA3, which was initially identified as a marker exclusive to PCa. These variations exhibit distinct expression patterns in malignant and normal prostate cells, indicating that PCA3’s role in PCa pathogenesis may be influenced by alternative splicing. Additionally, the functional implications of these splice variants might affect PCa cell proliferation, invasion, or response to androgen deprivation therapy by altering their binding to target molecules or subcellular localization (Lemos et al. 2019, Wang et al. 2014b).

Additional post-transcriptional regulation that can change the structure and sequencing of lncRNAs is known as RNA editing. For example, the lncRNA “metastasis-associated lung adenocarcinoma transcript 1” (MALAT1) is upregulated in PCa and linked to poor prognosis (Ahmadi-Balootaki et al. 2022, Yadav et al. 2023). These changes may affect the oncogenic characteristics of MALAT1 by altering its secondary structure and interactions with other molecules. The stability of lncRNAs,their capacity to interact with proteins, DNA, or other RNAs; and their regulatory roles in gene expression can all be altered by editing them in PCa (Ahmadi-Balootaki et al. 2022, Yadav et al. 2023).

The interaction between lncRNAs and microRNAs (miRNAs) represents a fascinating aspect of post-transcriptional regulation in PCa. Numerous studies have examined the functional association between HOTAIR and miRNAs in the initiation and advancement of PCa. Moreover, in castration-resistant PCa cell lines, HOTAIR was found to be upregulated by genistein, and when knocked down, cell invasion, proliferation, and migration were delayed, while apoptosis and cell cycle arrest were induced. Additionally, genistein directly targets HOTAIR, upregulating the tumor suppressor miR-34a, which in turn affects cell invasion, proliferation, and migration in PC3 and DU145 PCa cells. By introducing tri-methylation of H3K27 at the miR-193a promoter region, EZH2 combines with HOTAIR in PCa cells to mute the tumor suppressor miRNA miR-193a (Cantile et al. 2021).

Post-transcriptional regulation of lncRNA subcellular localization is critical for their function in PCa. Different cellular compartments provide distinct environments for lncRNA interactions and activities. For example, the lncRNA prostate cancer gene expression marker 1 (PCGEM1) is predominantly localized within the nucleus of PCa cells. This nuclear localization is essential for PCGEM1’s function in promoting androgen receptor-mediated gene activation, a key process in PCa progression (Wen et al. 2020). The mechanisms controlling lncRNA localization in PCa cells include interactions with specific RBPs, nuclear retention signals within the lncRNA sequence, and possibly RNA modifications. Understanding these localization patterns and their regulation can provide insights into lncRNA function and potential curative strategies in PCa.

## Future prospective and conclusions

LncRNAs play a key role in the control of gene expression at different levels, such as chromatin modification, transcription, and post-transcriptional processing. Moreover, they have been shown to have tumor-suppressive and carcinogenic properties. The application of lncRNAs in clinical practice is one of the most active fields of scientific inquiry. PCA is a rare but fatal disease with a poor prognosis. Unfortunately, there are not many choices for treating PCa. The treatments of PCa have few benefits in terms of survival; these include surgery, radiation, and chemotherapy. Comprehending the molecular pathophysiology of prostate tumors is crucial for improving therapeutic approaches. Improving PCa patient outcomes mostly requires early prediction and the ability to diagnose any metastases. To properly choose the appropriate course of treatment for PCa cases, diagnostic and prognostic biomarkers are required. The primary benefit of lncRNAs is their ability to be found in bodily fluids, which enables their usage as non-invasive indicators in clinical settings. Therefore, more research on circulating lncRNAs needs to be done in order to identify lncRNAs that might be employed in a practical sense as non-invasive PCa biomarkers. Notably, there were no trustworthy internal controls for lncRNA in serum or blood. Internal controls that are trustworthy are necessary to accurately estimate the levels of circulating lncRNA in the future. Overall, elucidating the mechanism of lncRNAs’ aberrant expression and the downstream mechanism of lncRNAs in malignancies will help us better understand lncRNA expression and will enable us to develop new tumor markers for clinical diagnosis and prognosis evaluation of tumors.

In conclusion, lncRNAs have critical roles in PCa initiation, progression, and metastasis through controlling various cellular processes such as proliferation, apoptosis, angiogenesis, and EMT. In particular, MALAT-1*,* CCAT2*,* DANCR, LncRNA-ATB, PlncRNA1, LincRNA-21, POTEF-AS1, ZEB1-AS1, SChLAP1, and H19 are key players in regulating the aforementioned processes. LncRNAs possess crucial clinical implications in PCa, as diagnostic and prognostic biomarkers, as well as medicinal targets. Comprehending the regulatory roles of lncRNAs in PCa predisposes to early detection, advanced assessment of severity, and designing innovative therapeutic plans. Targeting lncRNAs could pave the road for establishing personalized medicine. However, more studies are required to switch these findings into clinical practice. Moreover, various natural products have been shown to significantly influence the regulation of lncRNAs, displaying promise for cancer treatment and prevention. Eventually, targeting lncRNAs could pave the way for advancements in personalized medicine. Nevertheless, additional research is essential to convert these findings into practical clinical applications.

## Supplementary Information

Below is the link to the electronic supplementary material.

Supplementary file1 (PDF 2115 KB)

## Data Availability

All source data for this work (or generated in this study) are available upon reasonable request.
