# Supplementary material for: Natural products and long non-coding RNAs in prostate cancer: insights into etiology and treatment resistance
Source: Naunyn Schmiedebergs Arch Pharmacol. 2025 Jan 18;398(6):6349–68. doi: 10.1007/s00210-024-03736-x (PMC12125094; doi:10.1007/s00210-024-03736-x)
Supplement: Supplementary file 1 — Supplementary file1 (PDF 2115 KB) [file 210_2024_3736_MOESM1_ESM.pdf]

49 Spadina Ave. Suite 200
Toronto ON M5V 2J1 Canada
www.biorender.com

Confirmation of Publication and Licensing Rights

December 4th, 2024

Subscription Type: *Lab - Academic*
Agreement number: *FO27MKYD8G*
Publisher Name: *Naunyn-Schmiedeberg's Archives of Pharmacology*

Citation to Use: *Created with BioRender* *https://BioRender.com/y76v349*

To whom this may concern,

This document is to confirm that Hanan Elimam has been granted a license to use the BioRender Content, including icons, templates, and other original artwork, appearing in the attached Completed Graphic pursuant to BioRender's Academic License Terms. This license permits BioRender Content to be sublicensed for use in publications (journals, textbooks, websites, etc.).

All rights and ownership of BioRender Content are reserved by BioRender. All Completed Graphics must be accompanied by the following citation: "Created in BioRender. Ahms, A. (2024) https://BioRender.com/y76v349".

BioRender Content included in the Completed Graphic is not licensed for any commercial uses beyond use in a publication. For any commercial use of this figure, users may, if allowed, recreate it in BioRender under an Industry BioRender Plan.

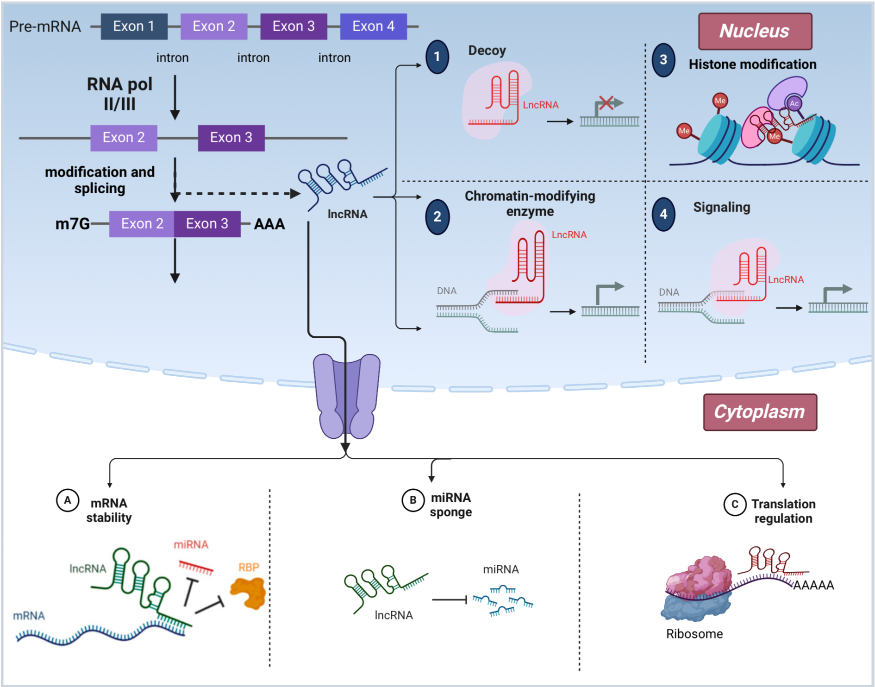

For any questions regarding this document, or other questions about publishing with BioRender, please refer to our BioRender Publication Guide, or contact BioRender Support at support@biorender.com.

49 Spadina Ave. Suite 200
Toronto ON M5V 2J1 Canada
www.biorender.com

Confirmation of Publication and Licensing Rights

December 4th, 2024

Subscription Type:	*Lab - Academic*
Agreement number:	*FE27MKXOK8*
Publisher Name:	*Naunyn-Schmiedeberg's Archives of Pharmacology*

Citation to Use: *Created with BioRender.* *https://BioRender.com/w07c483*

To whom this may concern,

This document is to confirm that Hanan Elimam has been granted a license to use the BioRender Content, including icons, templates, and other original artwork, appearing in the attached Completed Graphic pursuant to BioRender's Academic License Terms. This license permits BioRender Content to be sublicensed for use in publications (journals, textbooks, websites, etc.).

All rights and ownership of BioRender Content are reserved by BioRender. All Completed Graphics must be accompanied by the following citation: "Created in BioRender. Ahms, A. (2024) https://BioRender.com/w07c483".

BioRender Content included in the Completed Graphic is not licensed for any commercial uses beyond use in a publication. For any commercial use of this figure, users may, if allowed, recreate it in BioRender under an Industry BioRender Plan.

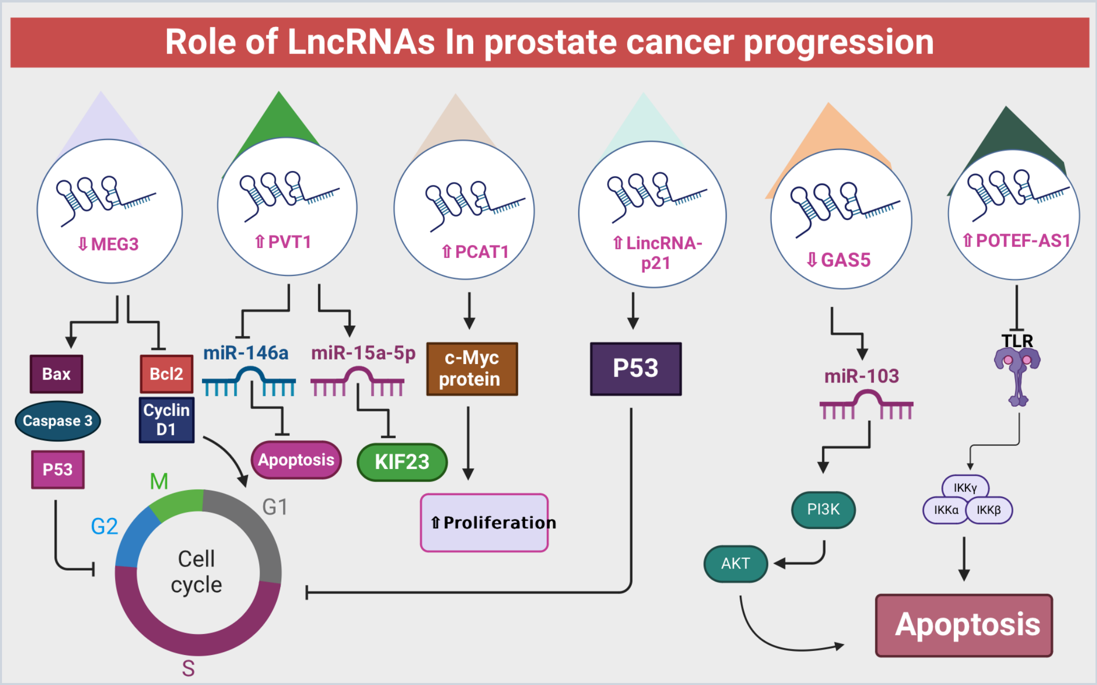

For any questions regarding this document, or other questions about publishing with BioRender, please refer to our BioRender Publication Guide, or contact BioRender Support at support@biorender.com.

49 Spadina Ave. Suite 200
Toronto ON M5V 2J1 Canada
www.biorender.com

Confirmation of Publication and Licensing Rights

December 10th, 2024

Subscription Type: *Lab - Academic*
Agreement number: *FW27NCWSZW*
Publisher Name: *Naunyn-Schmiedeberg's Archives of Pharmacology*

Citation to Use: *Created in BioRender. Ahms, A. (2024) https://BioRender.com/b66h402*

To whom this may concern,

This document is to confirm that Hanan Elimam has been granted a license to use the BioRender Content, including icons, templates, and other original artwork, appearing in the attached Completed Graphic pursuant to BioRender's Academic License Terms. This license permits BioRender Content to be sublicensed for use in publications (journals, textbooks, websites, etc.).

All rights and ownership of BioRender Content are reserved by BioRender. All Completed Graphics must be accompanied by the following citation: "Created in BioRender. Ahms, A. (2024) https://BioRender.com/b66h402".

BioRender Content included in the Completed Graphic is not licensed for any commercial uses beyond use in a publication. For any commercial use of this figure, users may, if allowed, recreate it in BioRender under an Industry BioRender Plan.

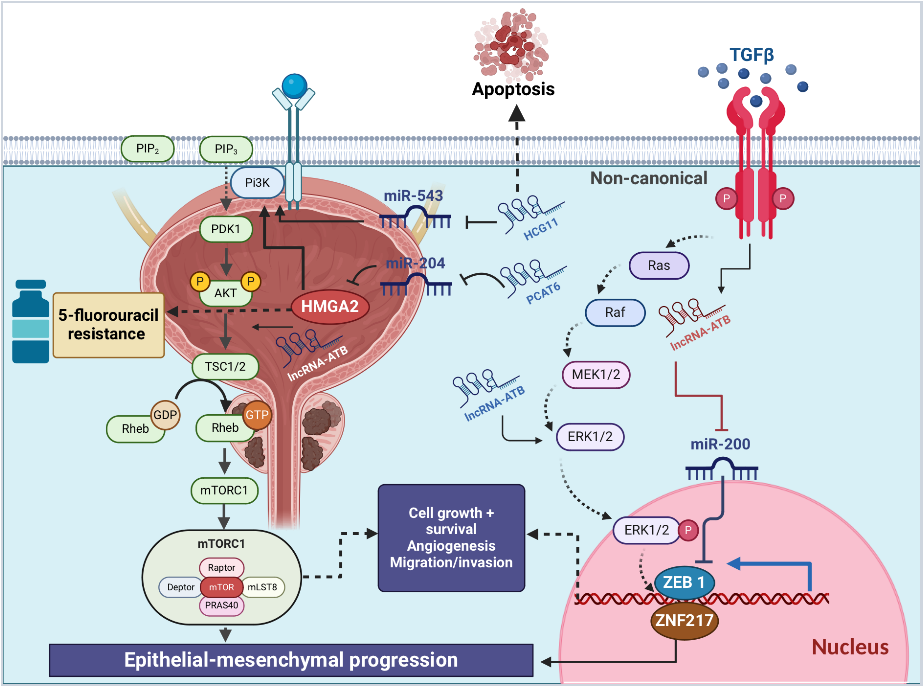

For any questions regarding this document, or other questions about publishing with BioRender, please refer to our BioRender Publication Guide, or contact BioRender Support at support@biorender.com.

49 Spadina Ave. Suite 200
Toronto ON M5V 2J1 Canada
www.biorender.com

Confirmation of Publication and Licensing Rights

December 4th, 2024

Subscription Type: *Lab - Academic*
Agreement number: *PW27MKZFK9*
Publisher Name: *Naunyn-Schmiedeberg's Archives of Pharmacology*

Citation to Use: *Created with BioRender* *https://BioRender.com/k48x291*

To whom this may concern,

This document is to confirm that Hanan Elimam has been granted a license to use the BioRender Content, including icons, templates, and other original artwork, appearing in the attached Completed Graphic pursuant to BioRender's Academic License Terms. This license permits BioRender Content to be sublicensed for use in publications (journals, textbooks, websites, etc.).

All rights and ownership of BioRender Content are reserved by BioRender. All Completed Graphics must be accompanied by the following citation: "Created in BioRender. Ahms, A. (2024) https://BioRender.com/k48x291".

BioRender Content included in the Completed Graphic is not licensed for any commercial uses beyond use in a publication. For any commercial use of this figure, users may, if allowed, recreate it in BioRender under an Industry BioRender Plan.

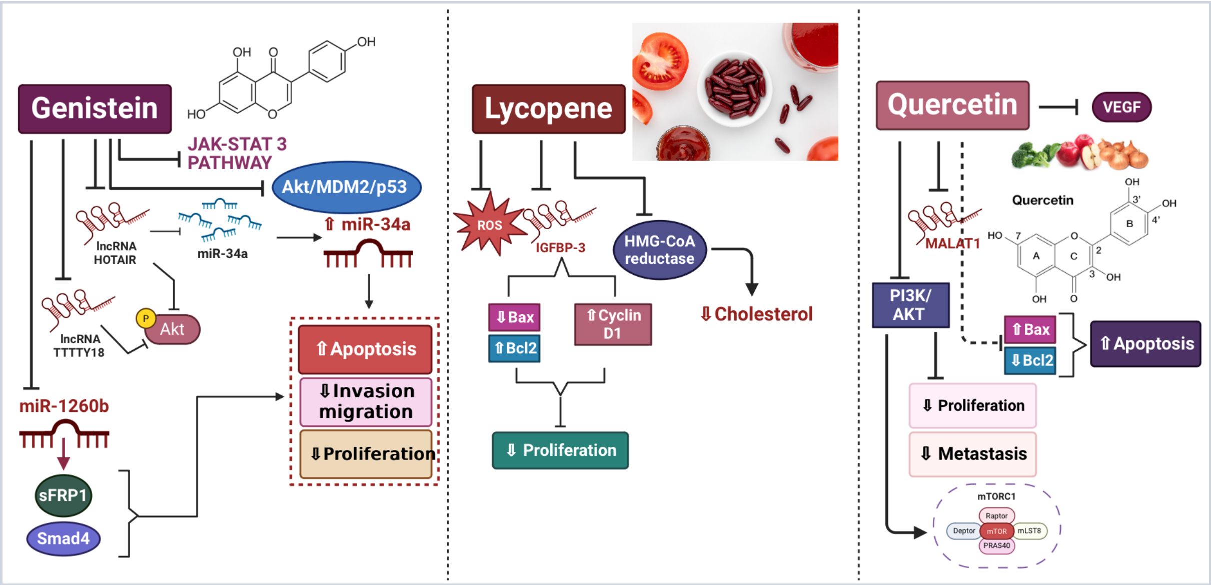

For any questions regarding this document, or other questions about publishing with BioRender, please refer to our BioRender Publication Guide, or contact BioRender Support at support@biorender.com.

49 Spadina Ave. Suite 200
Toronto ON M5V 2J1 Canada
www.biorender.com

Confirmation of Publication and Licensing Rights

December 4th, 2024

Subscription Type: *Lab - Academic*
Agreement number: *JN27MI4YC4*
Publisher Name: *Naunyn-Schmiedeberg's Archives of Pharmacology*

Citation to Use: *Created with BioRender.* *https://BioRender.com/t85p271*

To whom this may concern,

This document is to confirm that Hanim Elimam has been granted a license to use the BioRender Content, including icons, templates, and other original artwork, appearing in the attached Completed Graphic pursuant to BioRender's Academic License Terms. This license permits BioRender Content to be sublicensed for use in publications (journals, textbooks, websites, etc.).

All rights and ownership of BioRender Content are reserved by BioRender. All Completed Graphics must be accompanied by the following citation: "Created in BioRender. Ahms, A. (2024) https://BioRender.com/t85p271".

BioRender Content included in the Completed Graphic is not licensed for any commercial uses beyond use in a publication. For any commercial use of this figure, users may, if allowed, recreate it in BioRender under an Industry BioRender Plan.

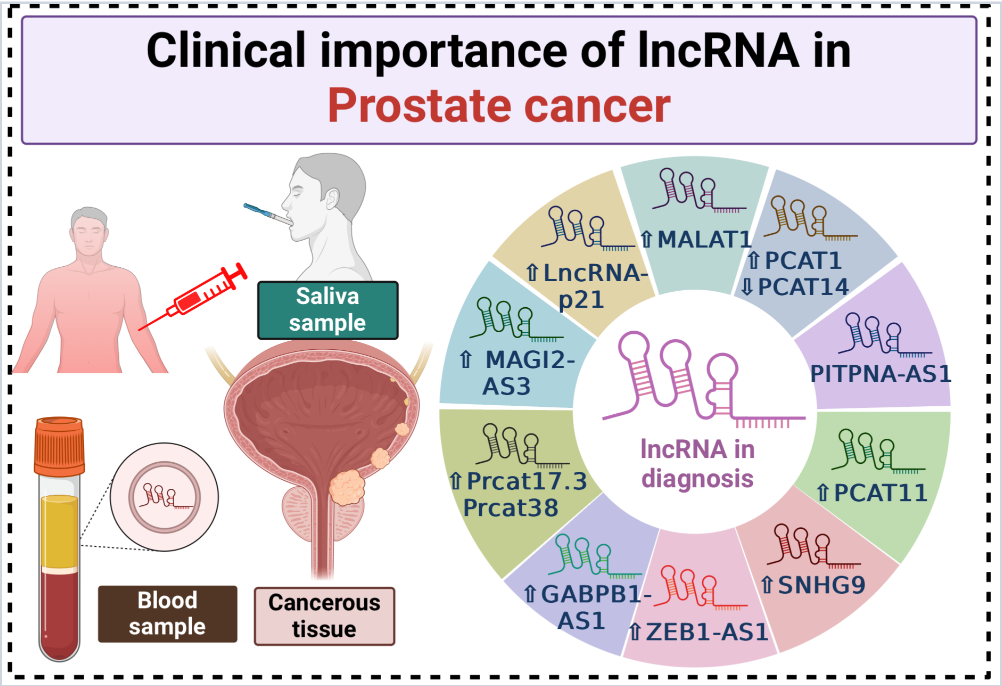

For any questions regarding this document, or other questions about publishing with BioRender, please refer to our BioRender Publication Guide, or contact BioRender Support at support@biorender.com.
